# Safety and Tolerability of SBRT after High-Dose External Beam Radiation to the Lung

**DOI:** 10.3389/fonc.2014.00376

**Published:** 2015-01-14

**Authors:** Dawn Owen, Kenneth R. Olivier, Limin Song, Charles S. Mayo, Robert C. Miller, Kathryn Nelson, Heather Bauer, Paul D. Brown, Sean S. Park, Daniel J. Ma, Yolanda I. Garces

**Affiliations:** ^1^Department of Radiation Oncology, Mayo Clinic Rochester, Rochester, MN, USA; ^2^Department of Radiation Oncology, University of Michigan, Ann Arbor, MI, USA; ^3^MD Anderson Cancer Center, Houston, TX, USA

**Keywords:** stereotactic body radiotherapy, reirradiation, external beam radiotherapy, lung cancer, toxicity tests, acute, late toxicity

## Abstract

**Purpose:** Stereotactic body radiotherapy (SBRT) is commonly used to treat unresectable lung nodules. Given its relative safety and effective local control, SBRT has also been used to treat recurrent lung nodules after high-dose external beam radiation (EBRT) to the lung. The toxicity of such treatment is unknown.

**Methods and Materials:** Between 2006 and 2012, 18 subjects at the Mayo Clinic with 27 recurrent lung nodules were treated with SBRT after receiving EBRT to the lung. Median local control, overall survival, and progression-free survival (PFS) were described. Acute toxicity and late toxicity (defined as toxicity ≥ and >90 days, respectively) were reported and graded as per standardized CTCAE 4.0 criteria.

**Results:** The median age of patients treated was 68 years. Fifteen patients had recurrent lung cancer as their primary histology. Twelve patients received ≥60 Gy of conventional EBRT prior to SBRT. SBRT dose and fractionation varied; the most common prescriptions were 48 Gy/4, 54 Gy/3, and 50 Gy/5 fractions. Only four patients had SBRT planning target volumes (PTVs) that overlapped more than 50% of their prior EBRT PTV. Two patients developed local recurrence following SBRT. With a median follow up of 21.2 months, median SBRT-specific overall survival and PFS were 21.7 and 12.3 months, respectively. No grade ≥3 acute or late toxicities were noted.

**Conclusion:** Stereotactic body radiotherapy may be a good salvage option for select patients with recurrent lung nodules following definitive EBRT to the chest. Toxicity is minimal and local control is excellent.

## Introduction

Intrapulmonary recurrence or new T1-2N0 lung primaries after definitive chemoradiation for locally advanced lung cancer is a major clinical dilemma. Data from randomized trials indicate locoregional recurrence may range from 30 to 40% with the majority being in-field failures (1, 2). Previous experience with lung reirradiation with conventional fractionated external beam radiation (EBRT) for recurrent disease yields suboptimal local control rates of 50–60% and 3–5% risk of grade 3 or higher toxicity (3). For patients with recurrence localized to peripheral and central locations, stereotactic body radiotherapy (SBRT) offers a potential salvage option, which has low toxicity and optimal local control.

One of the earliest reports of stereotactic salvage RT for intrapulmonary/mediastinal recurrence has been described for 17 patients treated to a median dose of 32 Gy in 8 fractions (4). Although the role of salvage RT was palliative, the crude local control rate was 70% (4). Since 2005, lung SBRT has become much more prevalent and higher hypofractionated doses are being prescribed with curative intent. A number of retrospective single institution reports suggest excellent local control rates exceeding 90% with limited follow up (5–8). The current study documents our institution’s experience with SBRT post high-dose chest EBRT with a median follow up of 21.2 months.

## Materials and Methods

Between 2006 and 2012, 18 patients with 27 recurrent lung nodules were treated with SBRT after receiving EBRT to the chest. Patient demographics including age, gender, tumor histology, chemotherapy use, EBRT prescription, SBRT dose, number of lung nodules treated, response to treatment, and acute and late treatment toxicity were collected. The data on SBRT treatment were collected in a prospective manner and additional information was obtained from retrospective chart review. Descriptive statistics were performed using JMP (Version 9.01, SAS Institute Inc., Cary, NC, USA).

Progression-free survival, overall survival, and follow up from the end of SBRT treatment were estimated using the Kaplan–Meier method. Progression-free survival (PFS) was defined as any local or distant progression following the end of SBRT treatment. Local failure was defined as in-field progression over serial CT-based imaging. Overall survival was calculated from the end of SBRT treatment. RECIST criteria were applied prospectively as scans became available to assess local control. This study was approved by the Mayo Clinic Institutional IRB ethics board.

External beam radiation and SBRT plans were designed using Eclipse (Varian, Palo Alto, CA, USA) treatment planning software. All patients underwent 4DCT planning scans. The internal target volume (ITV) was defined by contours on 10 phases of respiration. Expansion from ITV to planning target volume (PTV) for SBRT was 5 mm. Abdominal compression and breath-hold techniques were employed to minimize motion where necessary.

Prior to 2010, most patients had planning performed using 3DCRT and static field intensity-modulated radiation therapy (IMRT) techniques. After 2010, volumetric modulated arc therapy (VMAT) planning was more frequently used. We have found no treatment planning effect on local control or difference in toxicity among all lung SBRT patients (9). Daily, cone beam CT was used to verify the position of the ITV prior to each treatment delivery for SBRT.

While data were available in a prospectively collected Mayo Clinic SBRT database, all data were verified by retrospective chart review. Acute and late toxicity data were documented at every follow up in a prospective manner utilizing standard CTCAE version 4.03. Additional information was gathered from follow up notes and notes documenting effects during the treatment course.

## Results

### Patient, tumor, and treatment characteristics

**Figure 1:**
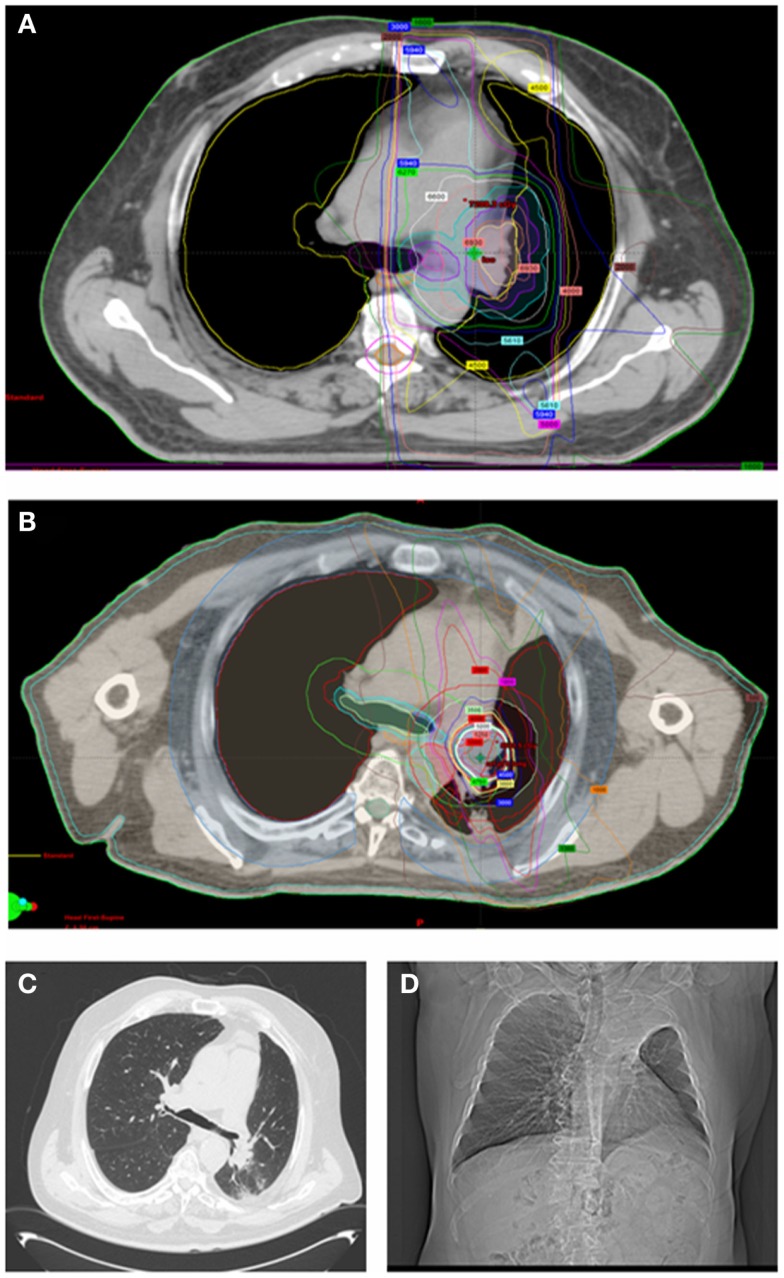
**Sixty-eight-year-old man with locally advanced non-small cell lung cancer (squamous cell histology) of the left upper lung treated with neoadjuvant chemotherapy followed by definitive chemoradiation (EBRT dose was 66 Gy/33 fractions to the left upper lobe, low paratracheal, and left hilar nodes)**. **(A)** shows his radical EBRT plan. He recurred 18 months later with an infield left hilar mass treated with 50 Gy/5 fractions using SBRT [**(B)** shows his SBRT plan]. Twelve months later, he remains free of recurrent disease but has collapse of the left upper lobe. He has no respiratory symptoms [**(C)** shows his most recent CT chest and **(D)** shows his chest X-ray at 8 months after SBRT].

The median age of patients was 68 years (range 20–76) and most patients were female (*n* = 11). Seventeen of the 18 patients included in the study had received prior definitive dose of lung EBRT with the most common prescription being 60 Gy/30 fractions. Of the 18 patients in the study, 14 patients were initially treated for locally advanced non-small cell lung cancer (Stage IIIA or IIIB; Table 1). PTV volumes for the initial EBRT ranged from 54 to 500 cc (median 254.7 cc). Four patients out of the 18 had SBRT PTV that overlapped significantly (>50%) with their prior EBRT PTV. Figure 1 shows an example of SBRT at a central site that had nearly 100% overlap with prior high dose EBRT field.

**Table 1:** **Demographics of lung SBRT patients (*N* = 18 patients; *N* = 27 SBRT courses)**.

**Median age (range in years) (*N* = 18)**	68 (20–76)
**Gender (*N* = 18)**
Male	7
Female	11
**Histology (*N* = 18)**
Sarcoma	2
Non-small cell lung cancer	14
Small cell lung cancer	1
Metastatic head and neck cancer	1
**Chemotherapy use within 1 month of SBRT (*N* = 18)**
Yes	3
No	15
**Stage of NSCLC prior to SBRT (*N* = 18)**
T1N1	1
T1N2	1
T2N1	3
T2N3	1
TXN2	2
T2N2	3
T4NX	1
T4N1	1
T4N2	1
Unknown	4
**Biopsy proven recurrence (*N* = 27)**
Yes	4
No	23
**Median (range) time from EBRT to SBRT (*N* = 18)**	18.4 months (1.5–112.8)
**Median (range) EBRT PTV Volume (*N* = 18)**	254.7 cc (54.0–500.2 cc)
**Prior EBRT prescription dose (*N* = 18)**
39 Gy/13#	1
45 Gy/30# (1.5 Gy BID)	1
48 Gy/12#	1
50 Gy/20#	1
50.4 Gy/28#	2
60 Gy/30#	7
64 Gy/32#	2
66 Gy/33#	2
70 Gy/35#	1
**Median EBRT dose (*N* = 18)**
BED10 (range 51 to 84 Gy_10_)	72 Gy_10_
EQD2 (range 42.3 to 70 Gy)	60 Gy
**Number of SBRT courses/patient (*N* = 18)**
1	12
2	5
3	1
**Median SBRT Tumor Size (*N* = 27)**	1.9 cm (0.5–4.94 cm)
**SBRT prescription dose (*N* = 27)**
40 Gy/5#	2
45 Gy/5#	1
48 Gy/4#	9
50 Gy/10#	1
50 Gy/5#	7
54 Gy/3#	6
60 Gy/3#	2
**Median SBRT dose (*N* = 27)**
BED10 (range 72 to 180 Gy_10_)	105.6 Gy_10_
EQD2 (range 60 to 150 Gy)	88 Gy
**Location of SBRT treatment (*N* = 27)**
Central	9
Peripheral	18
**SBRT location relative to EBRT (*N* = 27)**
Ipsilateral	17
Contralateral	10
**Response to treatment (based on imaging; *N* = 27)**
CR	6
PR	12
Progression	1
SD	8

Only four of the patients underwent biopsy to prove recurrence prior to SBRT (Table 1) with the remaining SBRT courses delivered for presumptive recurrence based on serial CT enlargement of a lung nodule and FDG avidity on PET–CT.

The median time between the end of EBRT and SBRT treatment was 18.4 months (range 1.5–112.8 months). The median follow-up period (from the end of SBRT to the time of last follow up) was 21.2 months (range 3.4–50.2 months). A total of 27 lung nodules were treated in 18 patients with SBRT (Table 1). The median tumor size and PTV volume treated with SBRT was 1.9 cm (range 0.5–4.94 cm) and 19.2 cc (6.4–79.6 cc), respectively. The most common dose prescriptions were 48 Gy/4, 50 Gy/5, and 54 Gy/3 fractions. More than half of the lesions treated with SBRT (17/27) were in the ipsilateral lung as the prior EBRT radiation field. Nine patients received SBRT for central lung tumors. Most patients only had one course of SBRT following EBRT (*n* = 13) and only three patients received chemotherapy following SBRT treatment. The three patients who received chemotherapy were continued on agents for lung adenocarcinoma, leiomyosarcoma, and Ewing sarcoma. All were with palliative intent as all three patients developed multiple lung metastases. Four patients received SBRT to tumors that lay within the previous EBRT high-dose region (>50% overlap of previous EBRT 50% isodose line) with one patient whose tumor recurred within the 95% isodose line.

### Dosimetry of cumulative EBRT and SBRT plans

Cumulative lung dose was calculated using sum plans using the Varian^®^ eclipse planning software. The cumulative mean lung volume irradiated in all patients was 3320 cc with a mean DC1000 cc (1000 cc of lung receiving a given dose or less) of 5.5 Gy and DC1500 cc of 10.5 Gy. Mean lung V5 was 60.6% (median 62.4%) and mean V20 was 29.5% (median 29.6%). The average mean cumulative lung dose was (median 17.8 Gy; range 6.0–26.6 Gy). The mean BED received by both lungs across cumulative EBRT and SBRT courses was 187.7 Gy_3_ (range 99.8–356.2 Gy_3_). The average mean heart dose was 21.6 Gy (median 23.6 Gy). Cumulative maximum dose to the esophagus and the chest wall ranged 38.9–78.4 Gy (median 62.5 Gy) and 53.5–150.5 Gy (median 93.7 Gy), respectively. Cumulative D4cc for bronchial tree ranged 20.1–79.6 Gy (median 57.1 Gy).

### Acute toxicity

Acute toxicity was any toxicity related to lung SBRT occurring within 90 days of the start of SBRT. Median time to the development of acute toxicity was 14.5 days (1–53 days range). Thirteen incidents of acute toxicity were documented in nine patients, which were either grade 1 or 2. No grade 3 or higher toxicities were noted. The most common toxicities were chest wall pain, nausea, and fatigue. No dosimetric or patient factors were predictors of acute toxicity. A summary of acute toxicities are noted in Table 2.

**Table 2:** **Acute toxicity (13 events in 9 patients)**.

Age (years)	Gender	Prior EBRT Dose	SBRT dose	SBRT courses	Time to toxicity	Grade + toxicity
63	Female	48 Gy/12 fx	48 Gy/4 fx	3	1 day	Grade 1 fatigue (counted as two separate events as patient experienced this event with two separate courses of SBRT)
60	Male	45 Gy/30 fx (1.5 Gy BID)	45 Gy/5 fx	1	1 day	Grade 1 nausea
71	Female	60 Gy/30 fx	54 Gy/3 fx	1	1 day	Grade 1 fatigue
64	Male	50.4 Gy/28 fx	54 Gy/3 fx	1	Unknown^a^	Grade 1 nausea
76	Female	50 Gy/20 fx	50 Gy/5 fx	1	1 day	Grade 1 cough
31	Male	60 Gy/30 fx	48 Gy/4 fx	1	Unknown^a^	Grade 1 dyspnea
					Unknown^a^	Grade 1 chest wall pain
71	Female	64 Gy/32 fx	48 Gy/4 fx	1	53 days	Grade 2 dyspnea
					53 days	Grade 2 chest wall pain
20	Male	50.4 Gy/28 fx	40 Gy/5 fx	2	33 days	Grade 1 chest wall pain (counted as two separate events as patient experienced this event with two separate courses of SBRT)
64	Male	60 Gy/30 fx	50 Gy/5 fx	1	Unknown^a^	Grade 1 nausea

*^a^For these four cases, the exact time to acute toxicity was unknown but the follow up notes by radiation oncology document these attributable effects <90 days from the end of treatment*.

### Late toxicity

Twelve late events (toxicity arising beyond 90 days from the end of SBRT) were documented in eight patients. The median time to the development of late toxicity was 176 days (range 88–493 days). All were either grade 1 or 2. The most common late effect was dyspnea. On univariate analysis, older age (*p* = 0.02) and chemotherapy use following SBRT (*p* = 0.02) were associated with late toxicity. None of the lung dosimetric parameters were predictive of late toxicity. The development of acute toxicity was not predictive of late toxicity. Site of tumor relative to prior EBRT volumes (ipsilateral/contralateral lung), central/peripheral location, and overlapping volumes were also not predictive of late toxicity. Table 3 summarizes the late toxicities. There were inadequate patients and events for multivariate analysis. Table 4 summarizes the univariate analyses for predictors of both acute and late toxicity.

**Table 3:** **Late toxicity (12 events in 8 patients)**.

Age (years)	Gender	Prior EBRT dose	SBRT dose	SBRT courses	Time to toxicity	Grade + toxicity
63	Female	48 Gy/12 fx	48 Gy/4 fx	3	314 days	Grade 1 dyspnea
60	Male	45 Gy/30 fx (1.5 Gy BID)	45 Gy/5 fx	1	158 days	Grade 2 cough
						Grade 2 dyspnea
71	Female	60 Gy/30 fx	54 Gy/3 fx	1	88 days	Grade 1 dyspnea
64	Male	50.4 Gy/28 fx	54 Gy/3 fx	1	175 days	Grade 2 radiation pneumonitis
76	Female	60 Gy/30 fx	54 Gy/3 fx	1	Unknown^a^	Grade 1 fatigue
70	Female	60 Gy/30 fx	48 Gy/4 fx	1	253 days	Grade 1 chest wall pain
					493 days	Grade 1 dyspnea
75	Female	64 Gy/32 fx	48 Gy/4 fx	1	Unknown^a^	Grade 1 cough
						Grade 1 dyspnea
70	Male	70 Gy/35 fx	48 Gy/4 fx	1	Unknown^a^	Grade 2 chest wall pain

*^a^ For these cases, the exact time to late toxicity was unknown but the follow up notes by radiation oncology document these attributable effects at greater than 90 days from the end of treatment*.

**Table 4:** **Univariate analysis of predictors of acute and late toxicity**.

Factor	Univariate analysis (*p* value) for acute toxicity	Univariate analysis (*p* value) for late toxicity
Age	0.79	0.02
Chemotherapy within 1 month of SBRT	0.58	0.02
Right/left lung location	0.15	0.89
Central or peripheral lesion	0.34	0.91
Ipsilateral/contralateral recurrence (compared to initial EBRT volume)	0.61	0.84
Time between EBRT and SBRT courses	0.54	0.87
Number of SBRT courses	0.81	0.88
BED10 of SBRT course	0.45	0.74
Gender	0.14	0.61

### Local control

Two of the 27 lesions recurred locally following salvage SBRT. One was a central lesion and the other was a peripheral lesion. Actuarial local control at 2 years was 90%. Given the very low failure rate, it was not possible to examine any factors that predicted for local failure.

### Overall survival and progression-free survival

Median SBRT OS (defined as survival from the date of completion of SBRT) was 21.0 months (range 0.7–47 months) and median SBRT PFS was 23.5 months (0.7–47 months). Actuarial 1 year OS from the end of SBRT was 88% and SBRT-specific PFS at 1 year was 58%.

## Discussion

The current study supports the potential use of lung SBRT as a salvage modality after high-dose chest radiotherapy. With a local control rate of 90% at 1 year and no grade 3 or higher toxicity noted, SBRT was very well tolerated in our cohort of patients.

To date, there have been very few published reports of SBRT treatment following high-dose chest EBRT. Coon et al. reported on 12 patients who received 60 Gy in 3 fractions to recurrent lung nodules post-EBRT treatment. With a median follow up of 12 months, no grade 3 or higher toxicity was reported and local control was 92% at 1 year. The PTV volume treated was relatively small (median of 14.3 cm^3^) (5, 10). The analysis of the retreated patients was not extensive as they were included among 51 patients who had received prior SBRT for solitary lung nodules.

Kelly et al. described the outcomes for 37 patients who received SBRT following conventional radiotherapy for locally advanced lung cancer (6). Their cohort received a single course of SBRT to a solitary targeted lesion (median GTV size 1.7 cm, range 0.6–3.8 cm) (6). Most patients (*n* = 24) had previously received 60 Gy or higher and were retreated to 50 Gy in four fractions (*n* = 26) (26/37). The dose fractionation was determined from their prior study in which patients with T1 lesions post-EBRT had better local control (100% at 2 years) with 50 Gy/4 fractions than 40 Gy/4 fractions (local control 50%) (11). Two patients had deliberate suboptimal SBRT coverage due to preservation of critical structures for in-field recurrences (defined as within the 30 Gy or 50% isodose line of the previous EBRT plan) (6). With a median follow up of 15 months, the crude local control at 2 years was 95%. PFS at 2 years post-SBRT was 59% with 74% of relapses occurring within the lungs (6). Similar to our study, their treatment was very well tolerated with no acute toxicity. However, at least one grade 3 late toxicity was reported in 33% of patients: radiation pneumonitis (*n* = 7), esophagitis (*n* = 3), skin ulcer (*n* = 2), and cough (*n* = 1) (6, 10). Radiation pneumonitis was not associated with in-field relapse. No grade 4 or 5 toxicities were noted.

An update of the MD Anderson experience was published in 2012 on 74 patients, indicating that the practice of using SBRT for new lung nodules or recurrent disease post-conventional chest EBRT (median EBRT dose was 63 Gy with range of 30–79.2 Gy) has been expanding (7). The focus of this study was on the incidence and risk factors for severe radiation pneumonitis. Local control remained excellent with only one patient experiencing in-field failure post-SBRT. With a median follow up of 16 months, the rate of grade 3 and above pneumonitis was 20.5% with one patient experiencing grade 5 pneumonitis. Risk factors associated with radiation pneumonitis included pre-SBRT performance status, the interval between EBRT and SBRT, ipsilateral versus contralateral treatment with SBRT relative to EBRT, a history of severe COPD (FEV1 <65% predicted), previous treatment to the bilateral mediastinum, and a cumulative V20 >39%. In the current study, no grade 3 and above toxicities were noted and no dosimetric parameters were associated with the development of any pulmonary toxicity (mostly grade 1 and grade 2 dyspnea). We did not routinely perform PFTs in the reirradiation setting. In our study, one-third of patients received more than one course of SBRT after conventional radiation to the chest.

Most recently, Memorial Sloan Kettering has reported on their experience of 39 patients (8). In this series, 22 patients had SBRT PTVs that abutted or overlapped the 50% isodose line of prior high-dose EBRT plans. There was a wide range of SBRT doses used with less than half the patients (15) receiving BED ≥100 Gy_10_. With median follow up of 12.6 months, local PFS was 77% at 1 year. This is much lower than in other published reports and in our current study likely reflecting the reduction in BED_10_ for SBRT dosing for post-EBRT in-field recurrences. Grade 3 and above toxicity was quite low with two cases of grade 3 pneumonitis, two cases of grade 3 chest wall pain, and one case of grade 4 skin toxicity. There were no grade 5 toxicities.

The current study shows excellent local control and very low toxicity even in patients who received more than one course of SBRT after conventionally fractionated high dose (≥60 Gy) EBRT to the chest. However, the study is limited by its small number of patients and its retrospective nature. Toxicity was not always collected prospectively although radiographic response was collected. Further, there was wide variability in the location of SBRT volumes post-EBRT with the majority of patients not having significant overlap of their previous EBRT PTV. As noted previously, only four patients in our study had >50% overlap between the SBRT and EBRT PTVs. This likely accounts for the low toxicity observed in our study. Our patient cohort also had much higher overall survival at 1 year (88%) than patients in other reports possibly indicating a selection bias of good performance oligometastatic or recurrent NSCLC patients.

While the few reports on reirradiation with SBRT following conventionally fractioned lung EBRT exist, they universally report high rates of local control. However, the risk for adverse effects, particularly severe radiation pneumonitis, is unclear. Studies in which patients were treated with lower BED had a lower risk of toxicity versus those in which most patients received high BED treatments. As SBRT becomes more widely applied for local recurrence and metachronous or synchronous lung nodules in patients who have been treated for locally advanced lung cancer, it will be important to assess the outcomes of such patients in a prospective clinical trial with a dose escalation component embedded in the trial design. Most recently, some institutions are exploring the option of SBRT as a primary modality for dose painting and localized boost after a lower dose of mediastinal or chest radiation (45–60 Gy) (12, 13). While these early reports indicate that this may be safe, guidance is needed to optimize or adapt SBRT dose prescriptions and delivery especially in patients who may have poor pre-existing lung function and significant overlap of their EBRT and SBRT PTVs.

## Conclusion

Stereotactic body radiotherapy can be an effective and safe salvage option for patients who have a recurrence locally or within the lung/mediastinum following high dose conventionally fractionated EBRT for locally advanced lung cancer. SBRT appears to be well tolerated but caution must be exercised as predictors of toxicity and optimal risk-adapted SBRT dose prescriptions for patients whose SBRT PTVs overlap their EBRT PTVs are not well understood due to the limited experience. This question should be studied in a prospective manner as the indications for lung SBRT continue to expand.

## Conflict of Interest Statement

The authors declare that the research was conducted in the absence of any commercial or financial relationships that could be construed as a potential conflict of interest.
